# Case Report: Rectal Perforation Secondary to a Toothbrush in an Elderly Man

**DOI:** 10.3389/fsurg.2022.921843

**Published:** 2022-06-10

**Authors:** Karthigesu Aimanan, Soon Yee Lim, Ahmad Junaidi Ahmad Hamidi, Tiong How Chieng, Firdaus Hayati

**Affiliations:** ^1^ Department of Surgery, Miri Hospital, Ministry of Health Malaysia, Miri, Sarawak, Malaysia; ^2^ Department of Surgery, Sibu Hospital, Ministry of Health Malaysia, Sibu, Sarawak, Malaysia; ^3^ Department of Surgery, Faculty of Medicine and Health Sciences, Universiti Malaysia Sabah, Kota Kinabalu, Sabah, Malaysia

**Keywords:** foreign bodies, pneumoperitoneum, radiography, rectum, case report

## Abstract

Rectal foreign bodies often constitute an arduous diagnosis and perplexing management. A 72-year-old gentleman who is mentally sound was brought to the emergency department for severe epigastric pain of a 1-week duration. On examination, he was pyrexial and in sepsis. The abdomen was guarded. A digital rectal examination was normal. Erect chest radiography revealed air under the diaphragm and abdominal radiography showed neither dilated bowel nor foreign body. A diagnostic laparoscopy was performed which revealed a yellow hard rod-shaped foreign body at the pelvis. Upon conversion to midline laparotomy, the foreign body was found to be a toothbrush with intraperitoneal rectal perforation of 1 cm in length. The brush was removed and the perforation was repaired primarily. A diverting transverse loop colostomy was created. Rectal foreign bodies may cause life-threatening rectal injuries including lacerations, bleeding, perforation, and obstruction. It is deemed crucial that any patient with rectal foreign body demands an orderly approach with the intention of diagnosis, management, and post-extraction evaluation.

## Background

Rectal foreign bodies often lead to a challenging diagnostic and management dilemma, starting from the initial evaluation in the emergency department and continuing through the post-extraction period. We report a rare case of rectal perforation secondary to a toothbrush in an elderly gentleman who was initially suspected of a perforated gastric ulcer. Based on the English literature review, up to date, there was only one case report on rectal perforation secondary to a toothbrush (1). We highlight a 72-year-old gentleman who presented with an acute abdomen and sepsis with pneumoperitoneum and we discuss our management plan.

## Case Presentation

**Figure 1:**
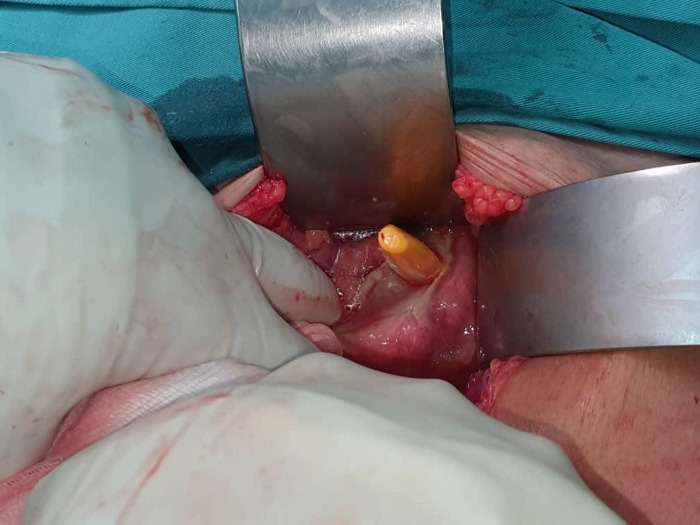
Rectal perforation by the tip of the toothbrush.

**Figure 2:**
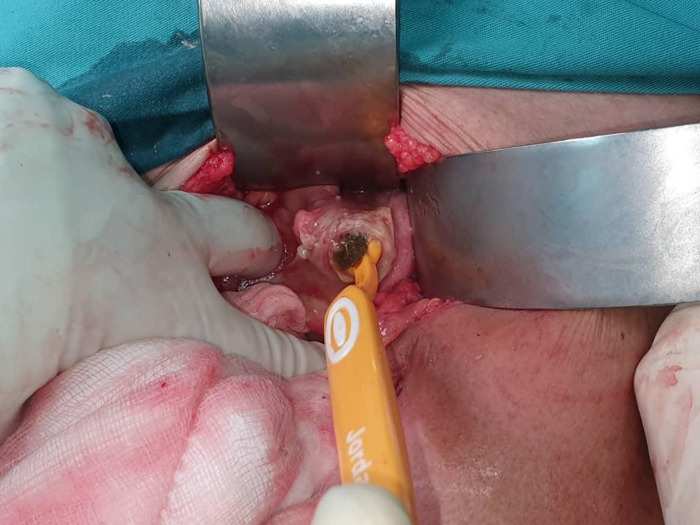
Extraction of the toothbrush from the rectum.

A 72-year-old gentleman was brought by family members for severe epigastric pain of 1-week duration. He is mentally sound and ADL independent prior to presentation. On examination, the abdomen was guarded. A digital rectal examination was normal. The patient is pyrexial and in sepsis. Erect chest radiography revealed air under the diaphragm and abdominal radiography showed neither dilated bowel nor foreign body. He then underwent a laparoscopy which revealed a yellow hard rod-shaped foreign body at the pelvis. The foreign body (Figures 1, 2) appeared to perforate the rectum.

Upon conversion to midline laparotomy, the foreign body was found to be a toothbrush. The rectal perforation was intraperitoneal with 1 cm in length. The brush was removed and the perforation was repaired primarily. In view of extensive contamination, a diverting transverse loop colostomy was created. The patient improved well on a postoperative day one and was allowed orally. In further history, he revealed a habit of using a toothbrush to assist in defecation and was unable to retrieve it back on one occasion. He did not reveal it to family members to avoid embarrassment. He was then referred to a psychiatrist for proper assessment. A colonoscopy after two months revealed no abnormality and he was planned for reversal of stoma.

## Discussion

Rectal foreign bodies are widely documented in literatures. It is possible to occur after self-insertion for anorectal disease, concealment, criminal assault or accidental causes, or (most commonly) for sexual purposes including sexual gratification and anal erotism (2). Various objects involved, including fruits and vegetables, light bulbs, bottles, impulse body spray cans, and sex devices that have been described as retained rectal foreign bodies. Since there numerous types of objects and the sequelae of trauma it can cause to local tissues of the rectum and distal colon, it is crucial to rely on a systematic approach to the diagnosis and management of rectal foreign bodies.

History taking in these patients might be misleading due to their embarrassment and reluctance to seek medical care. Moreover, in the emergency room, patients may often be dishonest on the reason for their visit, thus leading to extensive and unnecessary workups and further delays (3). Hence, routine imaging and laboratory investigations are crucial. An abdominal radiograph would be helpful to look for radioopaque objects by assessing their shape, nature, and location (4). However, for certain objects, they are radiolucent under x-ray such as in our case. A high index of suspicion is needed to accurately diagnose their condition. In addition, the utmost degree of professionalism is required by the attending physician in managing this condition (5).

A retained rectal foreign body can be categorised as either high- or low-lying relative to the rectosigmoid junction. A palpable object on a digital rectal examination is considered low-lying and may be considered for bimanual extraction at the bedside provided no evidence of perforation (6). High-lying retained rectal foreign body on the other hand usually necessitates endoscopic or surgical intervention. Several factors must be considered before making the decision between primary repair with or without colostomy in the surgical management of colorectal injuries. These include the site and cause of the injury, physiologic stability of the patient, and the use of antibiotic. Among all of these considerations in surgical management, the grade of colorectal injury has been most widely discussed (7). In this patient advanced age and peritoneal contamination lead us to decide on the diverting colostomy. Moreover, this is the safest approach for this patient because we were unsure of the nature of the injury distal to the perforation.

In conclusion, rectal foreign bodies can cause potentially fatal injuries which include lacerations, haemorrhage, perforation, and distal obstruction. A systematic approach is required for the diagnosis, treatment, and post-extraction assessment in a patient with rectal foreign body.

## Data Availability

The original contributions presented in the study are included in the article/Supplementary Material, further inquiries can be directed to the corresponding author/s.
